# Significant other reported outcome measures within European cohorts of severely injured patients: A systematic review

**DOI:** 10.1007/s00068-026-03286-2

**Published:** 2026-08-24

**Authors:** J. C. Van Ditshuizen, L. Van Den Berg, M. H. J. Verhofstad, P. H. C. Leliefeld, M. A. C. De Jongh, D. Den Hartog

**Affiliations:** 1 Trauma Research Unit Department of Surgery, Erasmus MC, University Medical Center Rotterdam, Rotterdam, The Netherlands; 2 Network for Emergency Care Southwest, Erasmus MC, University Medical Center Rotterdam, Rotterdam, The Netherlands; 3 Netwerk Acute Zorg Brabant, Elisabeth-Tweesteden Ziekenhuis, Tilburg, The Netherlands; 4 Department of Surgery, Elisabeth-Tweesteden Ziekenhuis, Tilburg, The Netherlands; 5 Tilburg School of Social and Behavioural Sciences, Tranzo, Tilburg University, Tilburg, The Netherlands

**Keywords:** Multiple trauma, Stress disorder, Post-traumatic, Burden, Caregiver, Quality of life, Relatives, Patient-reported outcome measures

## Abstract

**Purpose:**

Major trauma can have a long-term impact, leading to diminished health-related quality of life (HRQoL) for patients and their significant others (SOs). Patient reported outcome measures (PROMs) make this impact visible, providing expectation- and self-management. European studies assessing Significant Other Reported Outcome Measures (SOROMs) in cohorts of severely injured patients were reviewed.

**Methods:**

A systematic literature search was conducted. European studies from 2000 onwards assessing SOROMs in severely injured patient cohorts were included. Two reviewers independently screened all records, consensus was reached by a third reviewer.

**Results:**

2,909 studies were screened, resulting in 30 inclusions. Studies originated from Western and Northern Europe, overrepresented by traumatic brain injury (*n* = 27, 90%). Forty-three different SOROMs were identified. Twenty (67%) studies reported subacute and long-term reduction in HRQoL, and high prevalence (28–56%) of depression, anxiety, and stress within SOs. Outcomes of SOs and patients run parallel and are associated with coping strategies. Sixteen (53%) studies reported around 50% of unmet family needs (information and emotional support), and a disproportionate burden of care associated with loneliness and life satisfaction.

**Conclusion:**

SOs of severely injured patients reported reduced HRQoL, with high levels of depression, anxiety, and stress in the acute stage. Levels decline over time, but remain elevated in the long-term. Family bonding and social opportunities encourages secondary coping strategies, dampening mental issues and increasing patients’ HRQoL. Subacute and long-term interaction of SO and patient is crucial for rehabilitation and caregiver burden. The wide variety of SOROMs could benefit from a collateral standardisation with PROMs.

**Supplementary Information:**

The online version contains supplementary material available at 10.1007/s00068-026-03286-2.

## Introduction

In contemporary trauma care mortality is becoming less discriminative for quality of care [1–3]. As survival rates after severe injury have increased, trauma care professionals and public health care policies increasingly focused on patient-reported outcome measures (PROMs) [4–7]. Severe injuries can have great impact for the patient resulting in impaired quality of life, difficulties with return to work, health care consumption, post-traumatic stress, and social dysfunction [8–11]. Monitoring physical, psychological, and social health domains provides valuable insights during rehabilitation trajectories. These domains can support patients and trauma professionals in describing expected long-term outcomes and effective coping strategies.

Many severely injured patients will become (health-care) dependent, for a while or permanently. A big part of this dependency can be managed by an informal caregiver within the patients’ inner circle, a significant other (SO). This could be a family member like a parent or partner, but can also be covered by a sibling, a child, a (romantic) friend, an acquaintance, or neighbour. The interpersonal relationship of patient and SO can be under a lot of pressure for various reasons (e.g. financial stress, behavioural changes within the patient, the responsibility of care). Besides increased family needs and burden of care, psychological health status, health-related quality of life, and social functioning can be affected for significant others as well [12].

Parallel to PROMs, Family Reported Outcome Measures (FROMs) and proxy or relative versions of PROMs (proxROMs or PROMs-R) have been introduced [13, 14]. Many questionnaires (e.g. Impact Event Scale, Hospital Anxiety and Depression Scale, Short-Form Health Survey) lack validated relative or proxy versions, yet are administered to proxies or SOs. In proxy assessments, another person than the patient evaluates health related limitations. This is quite common for patients who are incapacitated due to cognitive impairments, in a palliative care setting, in paediatrics, or when a patient is not proficient in the language of available questionnaires. Without proxy reporting, relevant information may be lost. However, reliance on proxy responses introduces risk of proxy-bias [15].

Health-related outcomes of the social environment of the severely injured are underrepresented in trauma research. Nevertheless, reduced quality of life and life satisfaction among SOs have been reported [16]. Caregiving burden is associated with increased stress levels, a substantial proportion of families report unmet needs, and interpersonal relationships may become strained [17]. Coping strategies and behavioural challenges have a mediating effect on these domains [18]. This is especially relevant when personality traits or environment circumstances shift during life transitioning periods [19]. Significant others can be affected both in the acute phase and in the long-term. Meanwhile, they play a crucial role in patients’ rehabilitation trajectories and may contribute to post-traumatic growth. 

Recommendations on patient-centred outcome measures after major injury have been published previously [20]. At the same time, awareness should be raised for informal caregiver roles in rehabilitation trajectories of severely injured patients. Not all significant others are family related and many different health-related outcomes can be surveyed. Therefore, the current study introduces the term significant other reported outcome measures (SOROMs). This systematic review aims to provide an overview of SOROMs used in European cohorts of severely injured patients.

## Methods

### Search strategy

A systematic literature search was conducted across four electronic databases, including Medline AA, Embase, Web of Science Core Correlation, and CINAHL Plus. The search was performed in accordance with PRISMA guidelines and was registered in PROSPERO (CRD420251175098). A combination of relevant keywords and MeSH terms related to trauma, severe injury, PROMs, relatives, and European populations was used. All identified records were imported into Covidence^®^ to facilitate screening. Two reviewers (JVD and LVDB) independently screened titles, abstracts, and full text for eligibility. Disagreements were resolved by a third reviewer (MDJ). The final search was completed on October 14, 2025.

### Eligibility criteria

Studies were eligible for inclusion if they were conducted in Europe, published from 2000 onwards, and included cohorts of severely injured patients. Severe injury was defined as Injury Severity Score (ISS) ≥ 16, Glasgow Coma scale (GCS) ≤ 8, Intensive Care Unit (ICU) admission, spinal cord injury (SCI), traumatic limb amputations, or severe pelvic fractures. Studies were excluded if they did not assess SOROMs, focused on non-traumatic or combat-related injuries, or if the full text was unavailable. Questionnaires assessing coping and personality traits are neither PROMs nor SOROMs., Studies on these topics were not excluded from this review, and were considered an additional domain. This domain is intrinsically intertwined with health-related domains in which SOs can be affected, particularly when role and behavioural changes occur as a consequence of the traumatic event.

### Data extraction

Data extraction was performed using a standardised data extraction form in Covidence©. Data extraction included study characteristics (author, year of publication, country, study design, sample size), type of significant other, trauma subgroup, SOROMs used, and quantitative study results. Trauma subgroups were categorised according to injured body region, type of injury, and trauma mechanism, and were further dichotomised into paediatric and adult populations. All SOROMs used were categorised into three health domains: Health-related Quality of Life (HRQoL), psychological (anxiety, depression, post-traumatic stress), and social (family needs, burden of care). A fourth domain was added for personality traits (coping and interpersonal functioning).

### Quality assessment

Methodological quality and risk of bias of the included studies were assessed using a predefined checklist developed by the review team and implemented in Covidence^®^. The checklist consisted of commonly used quality and risk-of-bias domains for observational studies and tailored to SOROMs-based research. Items included completeness of outcome reporting, clarity of inclusion criteria, cohort specifics, adopted definition of severely injured, response rate and responders, recall bias, used SOROMs, pre-trauma comorbidities, and route of data collection. Each item was scored on low or high risk of bias. The percentage of items scoring low risk was calculated for each included study and for each item across all included studies.

### Synthesis of results

Each study was categorised according to health-related and personality-related domains. A brief summary was provided per study and synthesised across all included studies assessing that respective domain. Frequencies were accompanied with percentages, means with standard deviations, and median with P_25_-P_75_.

## Results

### Literature search

**Fig. 1:**
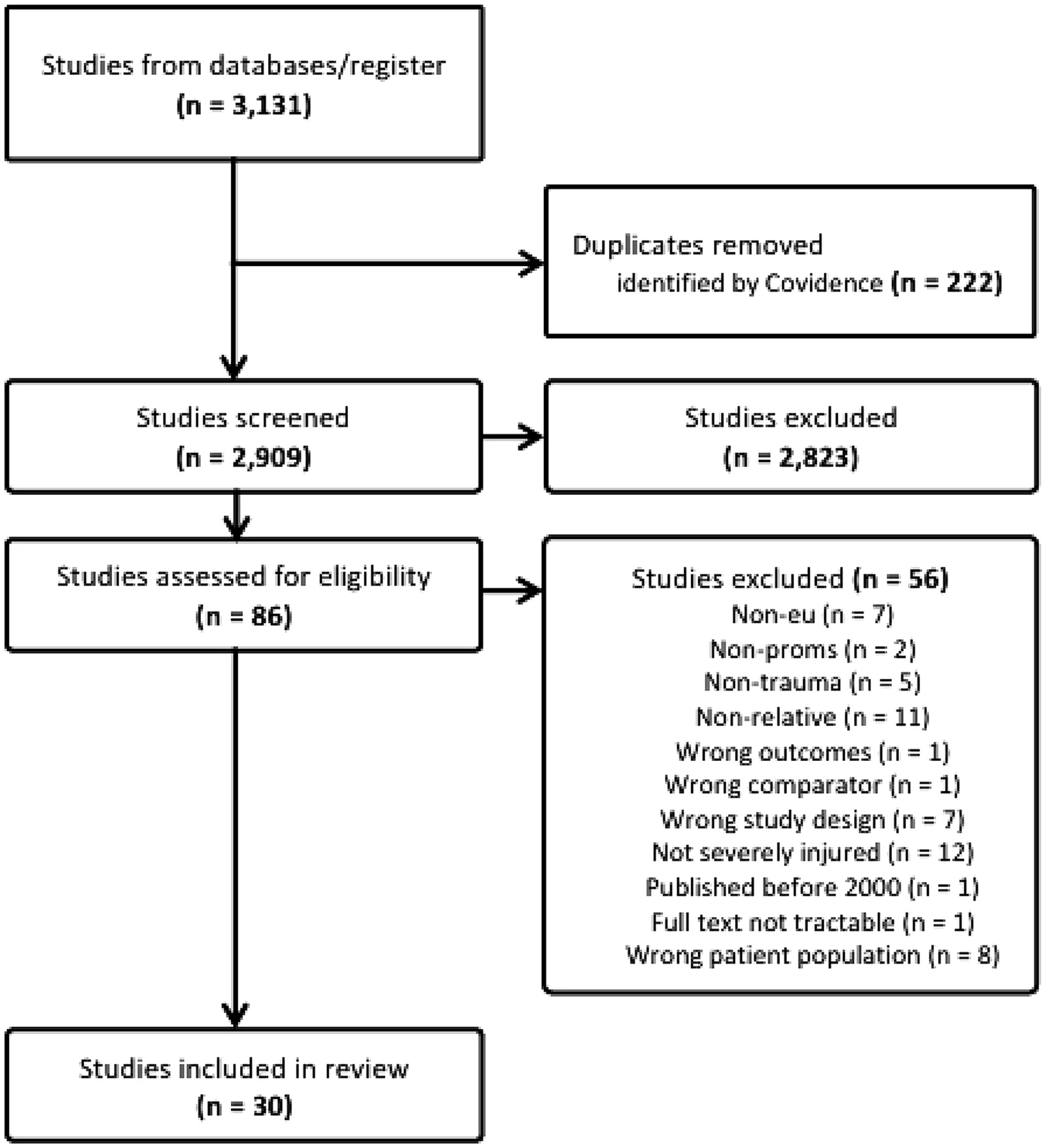
PRISMA flowchart of study inclusions

The search identified 3,131 potentially relevant studies. After removing 222 duplicates, 2,909 studies were screened on title and abstract. This resulted in exclusion of 2,823 studies, leaving 86 full-text studies assessed for eligibility, of which 30 were included. Main reasons for exclusion (*n* = 56) were no severe injuries, lack of SOROMs usage, inclusion of an ineligible patient population, or inappropriate study design. A Preferred Reporting Items for Systematic Reviews and Meta-Analysis (PRISMA) flow diagram is shown in Fig. 1.

### Baseline study characteristics

**Fig. 2:**
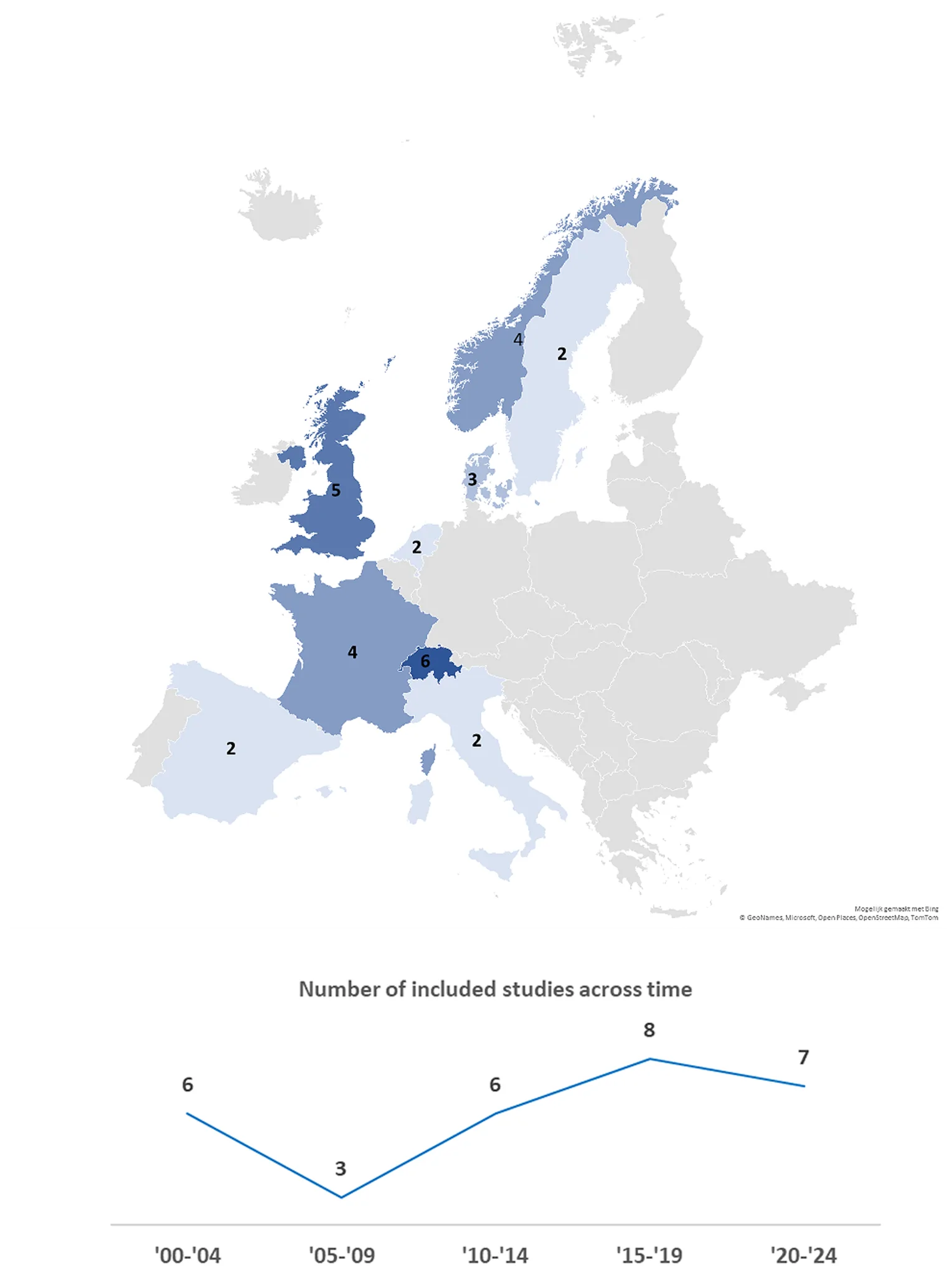
Geographic distribution of publications on SOROMs in severely injured trauma patient cohorts across Europe since 2000

Most included studies were conducted in Switzerland (*n* = 6, 20%), United Kingdom (*n* = 5, 17%), France (*n* = 4, 13%), and Norway (*n* = 4, 13%), (Fig. 2). The publication rate per five-years window was relatively stable (*n =* 3–8), with a drop between 2005 and 2009 (*n* = 3).

Table 1 displays that the majority of studies analysed TBI populations (*n* = 27; 90%). One study addressed road traffic accidents (RTA), one traumatic SCI, and one general severe trauma population. Five (20%) included studies were conducted in paediatric cohorts. Most studies were prospective cohort studies (*n* = 20, 67%), and the remaining 10 studies (33%) had a cross-sectional design. The overall sample size was 2,275 (median 82 *P*_*25*_*-P*_*75*_ 34–106).

**Table 1:** Overview of included studies reporting SOROMs in European cohorts of severely injured patients since 2000

Author	Country	Year	Injury	Patient Inclusion	Design	Sample size	Female (%) SO	SOROM	Follow-up	Conclusion
Patient population: Adults										
Hellawell et al. [33]	England	2001	TBI	GCS ≤ 12 GCS 13–15 & ISS ≥ 16	CSCS	70	-	Stress (VAS 10)	6.7y	Relatives’ stress was significant correlated with limitation in language, memory, physical, emotion, disturbed behaviour, social behaviour, subjective symptoms, and dependency. There is a high degree of similarity in the pattern of sequelae reported by carers.
Tyerman et al. [39]	England	2001	TBI	Severe TBI Rehab. program	CSCS	-	-	RSQ; FII; FHISDII; AFR; QII; FAD; PAIR GRIMS;	2.3y	Relatives are often strengthened in coping strategies by knowing available support services. Family ratings were less positively than the before the accident.
Watanabe et al. [32]	England	2001	TBI	GCS 3–8/9-12 JCS II/III Age 16–64	CSCS	18	67%	Family Stress; FNQ; FPSQ; FEAQ	2.7y	Family members living with TBI patients experienced problems. Appropriate rehabilitation services should be developed to help families as well as TBI patients.
Kozlowski et al. [21]	France	2002	TBI	GCS 3–8 < 24 h Age > 18	PCS	22	-	QOL-VAS (0–10)	3y	Patients’ QOL was discretely lower (5,48/10) than estimated by close relatives (5,91/10). Relatives’ QOL was similarly reduced (5,45/10). Behaviour and dependence in advanced activities influenced relatives’ QOL.
Inzaghi et al. [26]	Italy	2005	TBI	Admitted to rehabilitation centre	CSCS	16	75%	Interview	9.8y	Families felt isolated. Care-givers expressed steady worrying with effective coping to deal with depression (70%), mostly in patient dependency situations. Hardship levels were highest for working relatives and those close to the family. Happiness level is associated with disability.
Riley et al. [29]	Italy	2007	TBI	6 months − 1 year after trauma SO main carer	CSCS	40	78%	SDS; PSS; SSQ	1y	Depression and stress were associated with severe behaviour / less social support. Carer belief in own ability to control behaviour was associated with less stress. Carer belief in behaviour under patient control / hostile intentions associated with depression but less stress.
Van Baalen et al. [50]	Netherlands	2007	TBI	GCS 9–13/3-8 Age 18-65 Having a primary caregiver	PCS	51	67%	SIP-68; UCL; FAI	6 m-1y	The patients’ age and the caregivers’ coping style were independently associated with restrictions in participation. A passive coping style of the primary caregiver was negatively associated with the patient’s participation in society.
Pielmaier et al. [34]	Sweden	2011	TBI	MAIS head > 3 Admitted to neurosurgical facility	PCS	69	75%	IES-R	1 m	Clinically significant PTS symptoms were observed in 36 proxies (52.2%). Mean IES-R scores were significantly higher in women. More intrusion, avoidance and hyperarousal were associated with lower GCS.
Calvete et al. [31]	Spain	2012	TBI	Age > 17 Fluent Spanish Having a primary caregiver	CSCS	223	-	FNQ; TRIG; CES-D	8.2y	Secondary coping (e.g. acceptance/positivity) associated with less grief and depression. Primary coping (e.g. problem solving/emotional expression) and disengagement associated with more symptoms. Emotional and professional support affect coping.
Doser et al. [36]	Denmark	2014	TBI	Rehab. At TBI unit	CSCS	42	76%	FNQ	5.4y	Occupational and co-habitation were significantly affected. Health information had the highest importance and emotional support lowest importance. The five most important needs were met in 41%-50% of the families.
Lude et al. [22]	Switzerland	2014	SCI	SCI	PCS	55	-	WHO QOL-BREF	6w; 12w; 1y;2y	Besides the physical dimension, persons with SCI and their close persons seem to experience a similar change in QOL.
Manskow et al. [44]	Norway	2015	TBI	GCS 3-8 Unsedated < 24 h Age carer > 18	PCS	92	75%	CBS	1y	CBS was reported high in 16% and moderate in 34%. Lack of a social network, feeling loneliness, and patient’s functional status are predictors of all CBS indices. General strain, disappointment, and isolation were identified as areas in which caregiver burden is high.
Norup et al. [28]	Denmark	2015	TBI	GCS 3-8 Respiratory stable Age > 18	PCS	90	74%	SCL-90-R	entry; rehab. (start/end); 3 m/1y post discharge	Improvement was found in anxiety and depression after one year. Female had more depressive symptoms. Higher initial level of anxiety was associated with the patient being younger, having a lower level of function and consciousness, and SO being female or spouse.
Bayen et al. [43]	France	2016	TBI	GCS 3-8 Age > 15	PCS	98	81%	ZBI	4y	44% informal caregivers experience a heavy burden (multidimensional). Objective and subjective burden were associated with patient’s physical and mental disorders. Objective also with no co-residency status, and objective also with alcohol/drug abuse, and litigation involvement.
Haller et al. [23]	Switzerland	2017	TBI	< 24 h	PCS	140	79%	CISS SF-12	3 m; 6 m; 1y	Patients’ GOSE was associated with relatives’ mental SF-12 across age. For relative’s age > 50 years: Emotional coping negatively affect total and cognitive functioning. Avoidance negatively effect and task-oriented positively effect interpersonal functioning.
Haller et al. [48]	Switzerland	2017	TBI	Age > 15 MAIShead > 3 TC admitted	PCS	113	81%	MCS-s (LMS); PCRS-NR (relative)	3 m; 6 m; 1y	Interpersonal functioning decreased across time. For patient’s age ≤ 50: Mindful creativity positively affect personal functioning across time. Mindful creativity was associated with patients’ total and cognitive functioning.
Haller et al. [47]	Switzerland	2017	TBI	MAIShead > 3 TC admitted NACA > 3	PCS	130	79%	NEO-FFI	3 m; 6 m; 1y	Patients > 50: Agreeableness and extraversion affect interpersonal functioning. Extraversion affect functioning / cognition, mindful affect physical QOL. Patients < 51: Neuroticism affect neurological / emotional functioning, extraversion emotions / psyche.
Manskow et al. [16]	Norway	2017	TBI	Age > 17 Age SO > 15 GCS 3–8 < 25 h	PCS	119	77%	CBS; LS (Likert 5)	1y; 2y	30% of the families reported an increased burden, 15% a decrease from year 1 to 2. Loneliness was an independent predictor of increased burden from year 1 to 2. Life Satisfaction decreased from year 1 to 2.
Chesnel et al. [42]	France	2018	TBI	Age > 14 GCS 3–8	PCS	90	-	QOLIBRI; BICoQ	4y	Patients’ lack of awareness is associated with subjective burden perceived by a significant other. Patients’ lack of awareness was not associated with severity of injury, impairments, socio-demographics, mood disorders, return to work, or quality of life.
Anke et al. [37]	Norway	2020	TBI	Age > 15 Age SO > 18 GCS 3–8 unsedated < 25 h	PCS	110	78%	FNQ-R	1y; 2y	Family needs met at 1–2 years was 28%-55%. Family needs most met: health information. Family needs most unmet: emotional support. Predictive for unmet family needs: Severity of injury, age, female patients, spouses.
Gaertner et al. [49]	Switzerland	2020	TBI	MAIShead > 3 Aware/Motivated Fluent German, French, English Age > 16	PCS	166	83%	PCRS-NR (relative version)	3 m; 6 m; 1y	Interpersonal functioning between patient and relative was positively associated with the patients’: mental HRQoL, physical HRQoL over time among age > 50, GOSE among younger individuals.
Tsur et al. [24]	Switzerland	2020	TBI	MAIShead > 3 Age > 15 Fluent German, French, Italian Communicating	PCS	101	79%	IES-R; SF-12 (relative version); PCRS-NR	1y	Relatives’ physical health was predicted by relatives’ PTS. Relatives’ mental health was predicted by patients’ and relatives’ PTS. Relatives’ functioning level was predicted by patients’ PTS.
Stenberg et al. [38]	Sweden	2022	TBI	Age 18–65; GCS 3–8	CSCS	21	-	Interview	6.5 years	A sense of belonging, having a purpose and a social network are important within families. A family assessment will support families become involved in the process of rehabilitation.
Domensino et al. [45]	Netherlands	2023	TBI	ICU admitted; 6 m-5y after trauma; Age > 17	CSCS	29	79%	MDS-ABI (part D)	3.2 years	Informal caregivers generally felt competent to provide necessary care (81%). 31% experienced disproportionate caregiver burden. Injury severity not associated with outcomes on the MDS-ABI for patients and caregiver.
Mena-Marcos et al. [46]	Spain	2024	TBI	ICD-10 CM code S06.30	PCS	27	67%	Interview	5 m	Anticipation and fear prevailed in both groups, showing shared emotional patterns. Caregivers reported importance of staff interactions, misinformation, and psychological care. Lack of psychological support is a barrier to optimal patient and caregiver well-being.
Patient population: Children										
Tomlin et al. [41]	England	2002	TBI	ICU; Age 0–15	PCS	82	-	Family bonding	6w; 6 m; 1y	Parents did not feel prepared for the degree of help which their child required 1 year after discharge. Parents perceived that psychosocial family functioning deteriorated in a substantial number of families. Prevalence of this perception did not diminish over the study period.
Hawley et al. [27]	England	2003	TBI	Admitted ≥ 24 h Age 5–15	PCS	29	100%	PSI / SF; GHQ-12; PRS	1y	Related to problems amount, regardless of injury severity, parents suffered more stress than controls. Financial burden is related to TBI severity. One third of the parents reported poor psychological health. Stress and family burden may be alleviated by information and support.
Allenou et al. [35]	France	2010	RTA	Age 8–17	PCS	100	72%	PDI; PDEQ; PCL-S	5w	Parents perceived peritraumatic distress/dissociation comparable to trauma victims, with 18% of the mothers carrying a probable PTSD. Peritraumatic stress was predictive for PTSD.
Norup et al. [25]	Denmark	2013	TBI	GCS 3–9 (age < 6 or > 15) GCS 3–11 (age 5–15)	PCS	94	74%	SF-36	entry; start & end rehab.; 3 m & 1y post-disch.	Acute and sub-acute periods are an difficult time psychologically for many families. Family-based mental health interventions during acute and sub-acute phases are critical, especially for families around patients with severe deficits. Family QOL increased over time.
Hellstrom et al. [40]	Norway	2024	all	Age 0-18 NISS > 9	CSCS	38	-	FNQ	6 m	Families are in need of health information, emotional support, and care involvement. Low age and higher number of injuries were associated with more family needs.

AFRQII, Aylesbury Family Roles Questionnaire II; BAI, Beck’s Anxiety Inventory; BICoQ, Brain Injury Complaint Questionnaire; CBS, Caregiver Burden Scale; CES-D, Centre for Epidemiological Studies Depression Scale; CISS, Coping Inventory for Stressful Situations; CSCS, Cross-Sectional Cohort Study; FAD, Family Assessment Device; FAI, Functional Assessment Interview; FEAQ, Family Experiences and Attitudes Questionnaire; FHISDII, Family Head Injury Semantic Differential II; FII, Family Impact Interview; FNQ, Family Needs Questionnaire; FNQ-R, Family Needs Questionnaire relative version; FPSQ, Family Problems and Solutions Questionnaire; GCS, Glasgow Coma Scale; GHQ-12, General Health Questionnaire short version 12; GRIMS, Golombok Rust Inventory of Marital State; ICD-10 CM, International Classification of Diseases 10th Revision Clinical Modification; ICU, Intensive Care Unit; IES-R, Impact Event Scale Relative version; ISS, Injury Severity Score; LS, Life Satisfaction; MAIS, Maximum Abbreviated Injury Scale; MCS (LMS), Mindful Creativity Scale (Langer Mindfulness Scale); MDS-ABI, Minimal Dataset for persons with Acquired Brain Injury; NEO-FFI, Neuroticism Extraversion Openness Five-Factor Inventory; PAIR, Personal Assessment of Intimacy in Relation-ships; PCL-S, Posttraumatic stress disorder Checklist specific; PCRS-NR, Patient Competency Rating Scale for Neuro-rehabilitation; PCS, Prospective Cohort Study; PDEQ, Peritraumatic Dissociative Experiences Questionnaire; PDI, Peritraumatic Distress Inventory; PRS, Problem Resolution Scale; PSI-SF, Parenting Stress Index Short Form; PSS, Perceived Stress Scale; QOL, Quality Of Life; QOLIBRI, Quality Of Life After Brain Injury Scale; RSQ, Relative Screening Questionnaire; RTA, Road Traffic Accident; SCL-90-R, Symptom Checklist-90-Revised; SDS, Zung’s Self-Rating Depression Scale; SF-12, Short Form 12; SF-36, Short Form 36; SIP-68, Sickness Impact Profile-68; SO, Significant Other; SOROM, Significant Other Reported Outcome Measure; SSQ, Social Support Questionnaire; TBI, Traumatic Brain Injury; TC, Trauma Centre; TRIG, Texas Revised Inventory of Grief; UCL, Utrecht Coping List; VAS, Visual Analogue Scale; WHO QOL-BREF, World Health Organisation Quality Of Life Brief Version; ZBI, Zarit Burden Inventory

Table 2 displays the distribution of SOs, with parents (*n* = 744, 33%) and partners (*n* = 766, 34%) comprising the largest groups. In eight (27%) studies the relationship of the SOs completing the SOROM with the patient was not reported, reflecting 18% (*n* = 423) of all studies included. Fourteen (47%) studies reported whether the respondent was a primary caregiver, resulting in 1,032 (96%) surveyed primary caregivers. Twenty-one studies reported on sex of the SOs, resulting in 1,300 (78%) surveyed females. In six (20%) studies the whole family was interviewed, and in eight (27%) studies participants were qualitatively interviewed.

**Table 2:** Distribution of significant others and type of interviewing

	No. of studies (%)^^^	Sample size (%)
*Population*		
**Parent**	22 (73%)	744 (33%)
**Partner**	20 (67%)	766 (34%)
**Child**	16 (53%)	154 (7%)
**Sibling**	15 (50%)	106 (5%)
Other*	17 (57%)	82 (4%)
**Unknown**	8 (27%)	423 (18%)
**Total**	30 (100%)	2275 (100%)
*If reported*		
**Primary Caregiver**	14 (47%)	1032 (96%)
**Female**	21 (70%)	1300 (78%)
*Type of report - interview*		
**Patient/SO interviewed both**	15 (50%)	1045 (46%)
**Patient/SO interviewed separately**	11 (37%)	993 (44%)
**Qualitative interview**	8 (27%)	283 (12%)
**Family interview**	6 (20%)	188 (8%)
*Reported domain*		
**Health Related QoL**	8 (27%)	576 (25%)
**Psychological**		
** Anxiety**	1 (3%)	90 (4%)
** Depression**	3 (10%)	353 (16%)
** Stress**	8 (27%)	521 (23%)
**Social**		
** Family needs**	10 (33%)	603 (27%)
** Burden of care**	6 (20%)	455 (20%)
** Personality traits**	7 (23%)	863 (38%)

SO, Significant other; QoL, Quality of Life

^One study did not report sample size, and was considered zero

*Neighbour, extended family, (romantic) friend, colleague, acquaintance, etc

### Quality assessment

Table 3 presents the risk-of-bias per study, with higher scores indicating lower risk of bias. All studies combined scored a mean bias percentage of 82 (*sd* 7.2). The five most frequent items with a high risk of bias were: description of data-collecting personnel (57%), whether the cohort was part of a trauma registry (17%), recall bias (17%), pre-trauma physical health status (10%), and pre-trauma mental health status (7%).

**Table 3:**
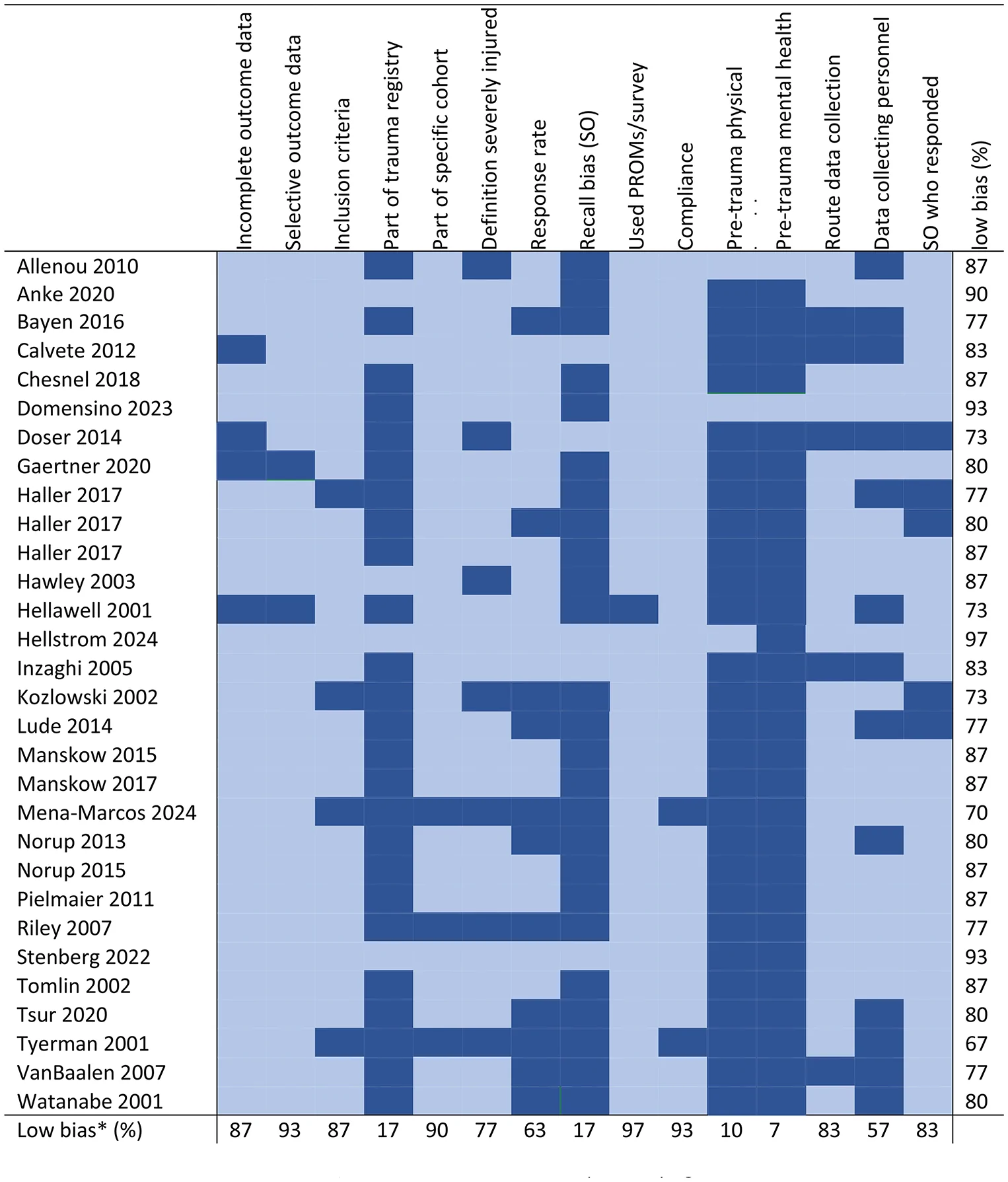
Quality assessment of included studies

*The light blue cells are scored a low risk bias, the dark blue cells are scored as potentially high risk bias

### Significant other reported outcome measures

A total of 43 different SOROMs were used across the included studies. HRQoL was reported in eight (27%) studies. In the psychological domain, eight (27%) studies reported on post-traumatic stress, three (10%) on depression, and one (3%) on anxiety. In the social domain, 10 (33%) studies reported on family needs and six (20%) on burden of care. Seven studies (23%) reported on personality traits and coping.

### Health related quality of life

All eight studies reported HRQoL of SOs to be associated with the patients’ health or social status. Two to three years post-trauma, the reduction of HRQoL among SOs paralleled those observed in patients across physical, mental, social, and environmental domains [21, 22]. The mental component summary score (SF-36) was associated with the patients’ GOSE and level of post-traumatic stress, as well as with post-traumatic stress of SOs [23–25]. The physical component summary score (SF-36) was predicted by post-traumatic stress of SOs as well [24]. Happiness and worrying in the SOs attributed to limitations of the patient, and was reported to be 2.94 (*sd* 1.18) and 2.88 (*sd* 1.41) on a five-point scale [26]. Nearly 70% of SOs experienced persistent worrying, 55% experienced tenderness in their relationship with the patient, nearly 50% felt nervous, and more than 40% reported feelings of sadness. Despite marked improvement in HRQoL, families experienced emotional distress and impaired HRQoL below population norms one year post-trauma [25]. Dissatisfaction with life increased from seven to 14% between the first and second year post-trauma [16]. Parental mental health was associated with the numbers of reported problems by them, alongside an increase in everyday psychological limitations from 18 to 35% in the first year post-trauma [27].

### Psychological limitations - anxiety and depression

From the patients’ admission to rehabilitation entry, 61% of the SOs scored above Danish population norms on anxiety levels, which decreased to 26% one year post-trauma. Depression levels above Danish population norms dropped from 54 to 23% one year post-trauma. In 51% of the responders, depression and anxiety were both above population norms at rehabilitation admission, decreasing to 18% one year post-trauma [28]. In the United Kingdom, levels of depression were higher than population norms, with 83% of primary caregivers reporting symptoms of at least a mild depression. Higher levels of depressive symptoms were significantly associated with beliefs that the patients’ behaviour was controllable or driven by negative intent [29]. Using the Center for Epidemiologic Studies Depression Scale, 29% of the caregivers scored 30 or higher, which is a severe depression indicator [30, 31]. Primary control coping strategies (e.g. emotion- and problem-focused) were associated with higher levels of depression, whereas secondary coping strategies (e.g. positive reappraisal and seeking social support) were associated with lower levels of depression and grief. Disengagement coping strategies (e.g. avoidance and wishful thinking) were associated with increased depression and grief. Support for involvement in care was associated with lower levels of depression. Coping functioned as a mediator between emotional or professional support and symptoms of depression and grief [31].

### Psychological limitations - stress

Danish families experienced emotional distress, particularly female spouses of younger patients with lower levels of consciousness and impaired daily functioning, who are more affected by distress in early phases of TBI rehabilitation [28]. In England, mean reported physical stress and mental stress were 2.5 (*sd* 2.1) and 2.7 (*sd* 1.1), on a scale of five. Both types of stress were related to opinions of others, outside and inside of the family [32]. On a scale of 10, the mean level of stress attributed to the patients’ TBI was 5.7 (*sd* 3.6) in a study from England [33]. In Italy, levels of stress were above population norms in a TBI cohort. Higher levels of stress were associated with patients’ difficult behaviour and less social support. Caregivers’ beliefs in their ability to control the patients’ behaviour were associated with lower levels of stress. The belief that the patients’ behaviour was controllable or motivated by hostile intent was associated with lower levels of stress [29]. In a Swiss TBI cohort, mean post-traumatic stress scores measured with the Impact Event Scale (relative version, IES-R) increased from three to six months and from six to 12 months post-trauma, remaining overall within the range of minimal to mild distress [24]. Mean IES-R sum scores were 13.4 (*sd* 7.3) for intrusion, 8.9 (*sd* 5.9) for avoidance, and 9.1 (*sd* 6.8) for hyperarousal. More than half of the respondents (*n* = 36, 52%) had an IES-R score of ≥ 33, indicating a clinical relevant symptoms of a post-traumatic stress disorder (PTSD) [34].

A large part of French mothers with an injured child due to a RTA reported peritraumatic stress (Peritraumatic Distress Inventory; *n* = 40, 55.6%). Dissociation was also frequently reported by these mothers (Peritraumatic Dissociative Experiences Questionnaire; *n* = 22, 30.6%). Probable PTSD among mothers was higher than among fathers (Posttraumatic stress disorder Checklist specific; *n* = 13, 18% vs. *n* = 1, 4%). Parental exposure to the traumatic event was associated with an increased risk of probable PTSD. No association was found between a history of prior traumatic events and the level of peritraumatic distress. However, distress and dissociation were positively correlated with PTSD symptoms. Maternal dissociation was correlated with higher levels of distress in fathers, however, probable PTSD in mothers was not associated with dissociative symptoms in fathers [35]. English parents of children with TBI were interviewed twice within two years after the traumatic event, with one year between the interviews. Part of the interview was the Parenting Stress Index Short Form. The proportion of parents scoring clinically relevant levels of total stress, indicating need for professional support, was high at both assessments (59% and 50%). Parental distress decreased between interviews from 28% to 19%, as well as difficulties with and problems of the child (from 59% to 39%). The presence of a dysfunctional parent-child interaction increased from 52 to 65%. In the control group, corresponding proportions ranged from 0% to 17%.

### Family needs

Studies from England, Denmark, Norway and Sweden identified health-related information as the most important need, which also demonstrated the highest percentage of needs being met [31, 32, 36–38]. The most family needs met were rated by 62–76% as very important, less than half of these needs were met (45–50%), and the percentage of needs met declined from 55 to 49% between year one and two post-trauma [36, 37]. Emotional and professional support were reported to be the least met needs (28%), and perceived to be among the least important needs [36, 37]. Emotional support was associated with more primary coping strategies and less disengagement coping strategies, both of which were mediators for symptoms of depression and grief. Instrumental support was associated with less primary coping strategies [31]. Interactions between family members and patient was worsened in 5% of cases, whereas 55% reported an improvement. Conversely, 61% reported concerns about others’ opinion (28% specifically regarding the opinions of other family members), alongside anxiety, tiredness, frustration, and lack of self-time as the main reported problems [32].

Findings from an English retrospective study on specialist family services and a Swedish seven-years post-trauma cohort corroborated these results, underlining the need for emotionally supportive family-centred communication to alleviate the burden, sense of loneliness and social isolation [38, 39]. A light touch and a flexible open door approach is most productive [39]. Inconsistently, a decline in community support network needs between one and two years post-trauma (44%-37%) has been described [37]. Such a decline in perceived support may contribute to increased stress, as lower levels of social support is associated with higher stress levels [29].

Within an English cohort of children, 10 families (35%) did not receive information provided by a hospital or rehabilitation centre. Of those who did, nine (47%) found the information helpful. The mean number of problems per family was 15.2 (*sd* 6.3), compared to 5.6 (*sd* 4.8) for control families. Twenty-two (76%) families reported emotional problems, 22 (76%) physical problems, 23 (79%) intellectual problems, 12 family problems (41%), and 16 (55%) other problems [27]. In a study from Norway most met needs were within the health information domain (*n* = 31, 82%), followed by involvement with care (*n* = 31, 82%). Least met needs were within domains emotional support (*n* = 27, 71%) and instrumental support (*n* = 22, 58%) [40].

In another English paediatric cohort, assessed from hospital discharge to one year post-trauma, 20% reported deterioration in their marriage or partnership, which was associated with worsened motivation and behavioural problems in children with TBI. One year post-trauma, 67% reported bonding between the primary caregiver carer and the child normalised, while 20% reported improved bonding. Sibling attitudes toward patients, and vice versa, worsened over time (from 12 to 38% and 40 to 46%). A deterioration in overall family bonding was also reported, increasing from 25 to 57% [41].

### Burden of care

A higher degree of unawareness of difficulties by the patient, as measured by the unawareness index by the Brain Injury Complaint Questionnaire, was associated with a greater subjective caregiving burden (Zarit Burden Inventory) [42]. A moderate to severe burden of care was reported by 11–34% [16, 42–45]. A French study reported a mean decrease in the ZBI between year one and four post-trauma, with both means in the clinical cut-off range from a mild to moderate burden of care (27 (20) to 21 (16)) [43]. Conversely, a Norwegian study observed increased caregiver burden as measured by the Caregiver Burden Strain, with 30% reporting an increase and 15% a decrease in scores [16]. The burden of care was associated with alcohol-drug abuse and litigation procedures, cognitive-behavioural limitations of the patient (measured by GOS-E and NRS-r), loneliness or no social network, disappointment, and life satisfaction [16, 43, 44]. In Spanish qualitative interviews caregiver overload was frequently mentioned in the acute, subacute, and chronic phases. Lack of psychological support is a barrier to optimal caregiver well-being [46]. A Dutch study found that 81% of informal caregivers felt competent to perform tasks associated with caring for the patient [45].

### Personality traits – mediating effect on significant others

Emotional and professional support were associated with symptoms of depression and grief via coping strategies as a mediator [31]. Emotional functioning improved as levels of agreeableness decreased, and stronger caregivers’ beliefs in their own ability to control the patients’ behaviour were associated with lower stress levels [29, 47]. The association of mindful creativity with interpersonal functioning significantly increased over time [48].

Primary and disengagement coping strategies were associated with more depression and more grief. However, grief was negatively correlated with several types of primary social support coping strategies. Secondary coping strategies were associated with less depression and grief [31, 47]. Total functioning decreased as primary emotion-focused coping strategies increased, interpersonal functioning increased as primary problem-focused coping strategies increased, and interpersonal functioning decreased as disengagement coping strategies increased [23].

### Personality traits – mediating effect on patient

The patients’ cognitive functioning decreased as primary emotion-focused coping strategies increased [23]. Mindful creativity showed a trend toward association with the patients’ interpersonal and cognitive functioning [48]. Interpersonal functioning was significantly associated with the patients’ mental HRQoL, independently of age.

For patients aged 50 years and older, the interaction between time and interpersonal functioning on patient recovery was positively significant [49]. Higher extraversion was linked to improved total, emotional, and cognitive functioning. Conversely, lower extraversion was associated with higher mental component scores. The patients’ neurological functioning improved with lower levels of neuroticism of the SOs, and the physical component score of the patient improved with more conscientiousness of the SOs.

For patients aged ≤ 50 years, emotional functioning improved with lower levels of neuroticism and extraversion. However, increased extraversion over time was associated with increases in mental HRQoL [47]. In the age group ≤ 50 years, mindful creativity was significantly associated with the patients’ overall functioning. This association was time independent [48]. Age and a passive coping style are independent related to being restricted in social participation [50].

## Discussion

Significant others of severely injured patients are affected across multiple outcome domains. Substantial and enduring impairments in HRQoL, psychological well-being, social participation, caregiving burden, and unmet family needs have been reported. Personality traits like coping strategies, are a mediator for the quality of interpersonal relationships [18]. A large part of SOs have troubles in multiple domains, as they are interconnected. Limitations have been reported in both the (sub)acute and the long-term phase of patients’ rehabilitation trajectories. Improvement over time is frequently observed and often run in parallel with the recovery curves observed among patients.

HRQoL is a multidimensional construct encompassing several health domains, typically addressed with one or a few questions per domain. Generic instruments may therefore be appropriate for SOs, as informal caregiving roles are often comparable across different patient conditions, regardless of specific illnesses. Some excluded studies combined trauma populations with acquired brain injury, non-trauma ICU admittance or non-traumatic SCI [51–56]. These patient groups have different diagnosis but follow a similar care pathways. Long-term effects like behavioural and cognitive changes within the patient may also be present in these patient groups. Sharing experiences of SOs across specialist care could be of added value. In addition, most studies reported health domains to be related. A generic questionnaire for significant others could therefore be of value as a screening or signalling tool for the patient as well as for the SOs themselves [4].

Depression, anxiety, and post-traumatic stress were reported with specific SOROMs. Across all studies, over half of SOs experienced clinically relevant symptoms of depression, anxiety and post-traumatic stress in the acute and subacute phases. Long-term prevalence rates were lower. Still, more than 20% continued to report clinically relevant symptoms, often with scores below population norms. Secondary coping strategies appeared to function as protective mediators between professional support and lower levels of distress. Whereas, primary control and disengagement coping strategies were associated with higher levels of distress [29, 47]. This was supported by findings showing increased distress among SOs perceiving a lack of control in response to the patients’ behavioural and cognitive limitations [29, 31]. Perceived loss of control was also associated with higher stress levels among parents of paediatric patients. Furthermore, maternal disengagement was related to higher levels of stress in fathers [35]. This may be related to socially constructed gender roles. Although, the fact that many parents do not cohabit or cohabit in a less traditional way should be considered when interpreting this association [57]. Troubled relationships and divorces are common, can be depressogenic and dysregulate the immune system [58].

Family needs and burden of care are more present when patients sustain more severe injury and exhibit greater cognitive and behavioural limitations [16, 44]. Health information was considered the primary family need and was also most frequently fulfilled. Substance abuse and litigation procedures place additional strain on interpersonal functioning and increases caregiver burden [43]. The included studies display contradictory findings regarding emotional support. Emotional support was deemed one of the least met needs, yet was also considered a low priority by caregivers. Nonetheless, feelings of isolation, disappointment, and reduced life satisfaction were associated with an increased caregiver burden [16, 44]. Loneliness and lack of social support were also reported to be associated with higher stress levels. This underlines recommendations to address interpersonal misunderstandings through emotional conversational support for the entire family [32, 39, 54]. Resilience may be strengthened by reduced sensitivity to others’ opinions [32]. Furthermore, fostering stronger family bonds and identifying more opportunities beyond the family context may promote secondary coping strategies. Subsequently, alleviating psychological distress in SOs and a potential benefit for patients’ HRQoL.

### Limitations & strengths

This review has several limitations to be addressed. All but three included studies comprised cohorts of TBI patients. All sample sizes were relatively small, and all cohorts were conducted in western European countries. This may limit the external validity and cross-cultural generalisability, particularly given that family-centred healthcare practices are shaped by the sociocultural context. A total of 53 outcome measures with 43 different SOROMs were used in the 30 included studies. Moreover, the lack of standardisation of SOROMs, comparable to PROMs, precluded quantitative synthesis through meta-analysis. Finally, most studies did not incorporate or adjust for physical and psychological baseline functioning, restricting the interpretation of post-traumatic changes.

Despite these limitations, this review provides a structured and comprehensive overview of the multidimensional impact of severe trauma on SOs. By integrating HRQoL, psychological functioning, caregiver burden, coping, and family needs, it offers a holistic perspective on non-fatal trauma care. This aligns well with the multidisciplinary nature of contemporary trauma systems and underscores the importance of systematically addressing health status and needs of SOs. Ultimately creating added value in rehabilitation trajectories of patients through a positive focus on SOs.

## Conclusion

Many SOs of severely injured patients experience substantial and often persistent reduction in HRQoL, frequently parallel to patient-reported outcomes. Although depression, anxiety, and stress levels decline after the acute phase, approximately one-fifth continues to report clinically relevant symptoms beyond one year post-trauma. Family dynamics and coping strategies play a critical role, since strong family bonding and engagement beyond family context are associated with secondary coping strategies, reducing psychological distress in SOs, and potentially improved patient HRQoL. The interaction between patient and SOs in the subacute and long-term phases is therefore crucial for rehabilitation outcomes and caregiver burden. Given the heterogeneity and limited comparability of existing PROMs and SOROMs in medical literature, further collateral standardisation is warranted to strengthen clinical implementation and future research.

## Supplementary Information

Below is the link to the electronic supplementary material.

Supplementary Material 1

## Data Availability

All data supporting the findings of this study are available within the paper and its Supplementary Information.
