# Supplementary material for: Significant other reported outcome measures within European cohorts of severely injured patients: A systematic review
Source: Eur J Trauma Emerg Surg. 2026 Aug 24;52(1):260. doi: 10.1007/s00068-026-03286-2 (PMC13503383; doi:10.1007/s00068-026-03286-2)
Supplement: Supplementary file 1 [file 68_2026_3286_MOESM1_ESM.docx]

**Supplements**

| **Database searched** | **Platform** | **Years of coverage** | **Records** | **Records after duplicates removed** |
| --- | --- | --- | --- | --- |
| Medline ALL | Ovid | 1946 - Present | 1245 | 1244 |
| Embase | Embase.com | 1971 - Present | 2026 | 1233 |
| Web of Science Core Collection* | Web of Knowledge | 1975 - Present | 813 | 196 |
| CINAHL Plus | EBSCO | 1982 - Present | 686 | 225 |
| Total | | | 4770 | 2898 |

*Science Citation Index Expanded (1975-present) ; Social Sciences Citation Index (1975-present) ; Arts & Humanities Citation Index (1975-present) ; Conference Proceedings Citation Index- Science (1990-present) ; Conference Proceedings Citation Index- Social Science & Humanities (1990-present) ; Emerging Sources Citation Index (2005-present)

No other database limits were used than those specified in the search strategies

Medline

(Multiple Trauma/ OR Accidents, Traffic/ OR Wounds, Penetrating / OR Wounds, Nonpenetrating/ OR Air Ambulances / OR ((Accidents/ OR exp Emergency Medical Services/) AND "Wounds and Injuries"/) OR Brain Injuries, Traumatic/ OR Amputation, Traumatic/ OR Injury Severity Score/ OR (((multipl* OR severe* OR massive* OR major OR penetrat* OR blunt*) ADJ3 (trauma* OR injur* OR wound* OR burn*)) OR (trauma* ADJ3 amputat*) OR multitrauma* OR multiinjur* OR multi-trauma* OR multi-injur* OR polytrauma* OR polyinjur* OR poly-trauma* OR poly-injur* OR barytrauma* OR baryinjur* OR bary-trauma* OR bary-injur* OR ((traffic* OR car OR motorcycle* OR injur* OR emergen*) ADJ3 accident*) OR (helicopter* ADJ3 (trauma* OR emergenc*)) OR (brain ADJ3 trauma*) OR neurotrauma* OR neuro*-trauma*).ab,ti,kw. OR (traum* ADJ3 (injur* OR wound*)).ti.) AND (Patient Reported Outcome Measures/ OR Quality of Life/ OR * Return to Work/ OR * Anxiety/ OR * Fear/ OR * Stress Disorders, Post-Traumatic/ OR * Psychological Well-Being / OR * Mental Health/ OR ((Return to Work/ OR Anxiety/ OR Fear/ OR Stress Disorders, Post-Traumatic/ OR Psychological Well-Being / OR Mental Health/ OR exp Family/ OR exp Social Environment/ OR Caregivers/) AND ("Surveys and Questionnaires"/ OR Self Report/)) OR (((patient* OR self OR relatives OR family OR parent* OR dependent* OR dependant* OR social-environment* OR guardian* OR social-network* OR social-circle* OR caregiver* OR carer OR carers) ADJ3 report* ADJ3 outcome*) OR prom OR proms OR (quality ADJ3 life) OR qaly* OR short-form OR shortform OR sf8 OR sf12 OR sf20 OR sf36 OR sf-8 OR sf-12 OR sf-20 OR sf-36 OR ((return-to-work* OR anxiety OR anxious* OR fear OR stress* OR ptsd OR wellbeing OR well-being OR mental-health OR psycholog*) ADJ10 (self-report* OR questionnaire* OR survey* OR assess*)) OR ((relative* OR famil* OR parent* OR dependent* OR dependant* OR social-environment* OR guardian* OR social-network* OR social-circle* OR caregiver* OR carer OR carers) ADJ6 (self-report* OR questionnaire* OR survey* OR assess* OR report*))).ab,ti,kw.) AND (exp Cohort Studies/ OR Controlled Before-After Studies/ OR Cross-Sectional Studies/ OR Registries/ OR (cohort* OR longitudinal* OR prospective* OR retrospective* OR cross-section* OR ((follow-up* OR outcome*) ADJ3 long*-term*) OR ((national* OR population* OR general*) ADJ3 survey*) OR (control* ADJ3 stud*)).ab,ti,kw. OR (follow-up* OR long*-term* OR register* OR registry* OR registries*).ti.) AND (exp Europe/ OR (Europe* OR Albania* OR Andorr* OR Armenia* OR Austria* OR Azerbaij* OR Balkan* OR Baltic-States* OR Estonia* OR Latvia* OR Lithuania* OR Belarus* OR Belgi* OR Benelux* OR Bosnia* OR Herzegovin* OR Bulgaria* OR Channel-Island* OR Croatia* OR Czech* OR England* OR France* OR French* OR German* OR Gibraltar* OR Britain* OR British* OR Greece* OR Greek* OR Hungar* OR Ireland* OR Irish* OR Isle-of-Man* OR Ital* OR Kosov* OR Liechtenstein* OR Luxembourg* OR Malta* OR Maltese* OR Moldova* OR Monac* OR Montenegr* OR Netherland* OR Dutch* OR Poland* OR Polis* OR Portug* OR Macedonia* OR Romania* OR Russia* OR San-Marin* OR Scandinavia* OR Denmark* OR Danish* OR Faroe* OR Finland* OR Finnish* OR Iceland* OR Norway* OR Norweg* OR Sweden* OR Swedish* OR Scotland* OR Scottish* OR Serbia* OR Slovakia* OR Slovenia* OR Spain* OR Spanish* OR Switzerland* OR Swiss* OR Ukraine* OR United-Kingdom* OR Vatican* OR Wales* OR Welsh* OR (Georgia* NOT (usa OR united-states))).ab,ti,kw.) NOT (news OR congres* OR abstract* OR book* OR chapter* OR dissertation abstract*).pt.

Embase

('multiple trauma'/de OR 'traffic accident'/exp OR 'penetrating trauma'/de OR 'blunt trauma'/de OR 'helicopter emergency medical service'/de OR ((accident/de OR 'emergency health service'/de) AND injury/de) OR 'traumatic brain injury'/de OR 'traumatic amputation'/de OR 'injury severity'/mj OR 'injury scale'/mj OR (((multipl* OR severe* OR massive* OR major OR penetrat* OR blunt*) NEAR/3 (trauma* OR injur* OR wound* OR burn*)) OR (trauma* NEAR/3 amputat*) OR multitrauma* OR multiinjur* OR multi-trauma* OR multi-injur* OR polytrauma* OR polyinjur* OR poly-trauma* OR poly-injur* OR barytrauma* OR baryinjur* OR bary-trauma* OR bary-injur* OR ((traffic* OR car OR motorcycle* OR injur* OR emergen*) NEAR/3 accident*) OR (helicopter* NEAR/3 (trauma* OR emergenc*)) OR (brain NEAR/3 trauma*) OR neurotrauma* OR neuro*-trauma*):ab,ti,kw OR (traum* NEAR/3 (injur* OR wound*)):ti) AND ('patient-reported outcome'/de OR 'quality of life'/exp OR 'quality of life assessment'/exp OR 'mood disorder assessment'/exp OR 'anxiety assessment'/exp OR 'return to work'/mj OR anxiety/mj/exp OR fear/mj OR 'mental stress'/mj/exp OR 'posttraumatic stress disorder'/mj OR wellbeing/mj/exp OR 'mental health'/mj/exp OR (('return to work'/de OR anxiety/exp OR fear/de OR 'mental stress'/exp OR 'posttraumatic stress disorder'/de OR wellbeing/exp OR 'mental health'/exp OR 'psychological aspect'/de OR family/de OR ‘family decision making’/de OR ‘social environment’/de OR Caregiver/exp) AND ('health survey'/de OR questionnaire/exp OR 'self report'/de)) OR (((patient* OR self OR relative* OR famil* OR parent* OR dependent* OR dependant* OR social-environment* OR guardian* OR social-network* OR social-circle* OR caregiver* OR carer OR carers) NEXT/3 report* NEAR/3 outcome*) OR prom OR proms OR (quality NEAR/3 life) OR qaly* OR short-form OR shortform OR sf8 OR sf12 OR sf20 OR sf36 OR sf-8 OR sf-12 OR sf-20 OR sf-36 OR ((return-to-work* OR anxiety OR anxious* OR fear OR stress* OR ptsd OR wellbeing OR well-being OR mental-health OR psycholog*) NEAR/10 (self-report* OR questionnaire* OR survey* OR assess*)) OR ((relative* OR famil* OR parent* OR dependent* OR dependant* OR social-environment* OR guardian* OR social-network* OR social-circle* OR caregiver* OR carer OR carers) NEAR/6 (self-report* OR questionnaire* OR survey* OR assess* OR report*))):ab,ti,kw) AND ('cohort analysis'/de OR 'longitudinal study'/de OR 'prospective study'/de OR 'retrospective study'/de OR 'follow up'/de OR 'controlled study'/de OR 'cross-sectional study'/de OR register/mj OR (cohort* OR longitudinal* OR prospective* OR retrospective* OR cross-section* OR ((follow-up* OR outcome*) NEAR/3 long*-term*) OR ((national* OR population* OR general*) NEAR/3 survey*) OR (control* NEAR/3 stud*)):ab,ti,kw OR (follow-up* OR long*-term* OR register* OR registry* OR registries*):ti) AND (Europe/exp OR (Europe* OR Albania* OR Andorr* OR Armenia* OR Austria* OR Azerbaij* OR Balkan* OR Baltic-States* OR Estonia* OR Latvia* OR Lithuania* OR Belarus* OR Belgi* OR Benelux* OR Bosnia* OR Herzegovin* OR Bulgaria* OR Channel-Island* OR Croatia* OR Czech* OR England* OR France* OR French* OR German* OR Gibraltar* OR Britain* OR British* OR Greece* OR Greek* OR Hungar* OR Ireland* OR Irish* OR Isle-of-Man* OR Ital* OR Kosov* OR Liechtenstein* OR Luxembourg* OR Malta* OR Maltese* OR Moldova* OR Monac* OR Montenegr* OR Netherland* OR Dutch* OR Poland* OR Polis* OR Portug* OR Macedonia* OR Romania* OR Russia* OR San-Marin* OR Scandinavia* OR Denmark* OR Danish* OR Faroe* OR Finland* OR Finnish* OR Iceland* OR Norway* OR Norweg* OR Sweden* OR Swedish* OR Scotland* OR Scottish* OR Serbia* OR Slovakia* OR Slovenia* OR Spain* OR Spanish* OR Switzerland* OR Swiss* OR Ukraine* OR United-Kingdom* OR Vatican* OR Wales* OR Welsh* OR (Georgia* NOT (usa OR united-states))):ab,ti,kw) NOT ([conference abstract]/lim AND [2000-2022]/py)

Web of science

(TS=(((multipl* OR severe* OR massive* OR major OR penetrat* OR blunt*) NEAR/2 (trauma* OR injur* OR wound* OR burn*)) OR (trauma* NEAR/2 amputat*) OR multitrauma* OR multiinjur* OR multi-trauma* OR multi-injur* OR polytrauma* OR polyinjur* OR poly-trauma* OR poly-injur* OR barytrauma* OR baryinjur* OR bary-trauma* OR bary-injur* OR ((traffic* OR car OR motorcycle* OR injur* OR emergen*) NEAR/2 accident*) OR (helicopter* NEAR/2 (trauma* OR emergenc*)) OR (brain NEAR/2 trauma*) OR neurotrauma* OR neuro*-trauma*) OR TI=(traum* NEAR/2 (injur* OR wound*))) AND (TS=(((patient* OR self OR relatives OR family OR parent* OR dependent* OR dependant* OR social-environment* OR guardian* OR social-network* OR social-circle* OR caregiver* OR carer OR carers) NEAR/2 report* NEAR/2 outcome*) OR prom OR proms OR (quality NEAR/2 life) OR qaly* OR short-form OR shortform OR sf8 OR sf12 OR sf20 OR sf36 OR sf-8 OR sf-12 OR sf-20 OR sf-36 OR ((return-to-work* OR anxiety OR anxious* OR fear OR stress* OR ptsd OR wellbeing OR well-being OR mental-health OR psycholog*) NEAR/10 (self-report* OR questionnaire* OR survey* OR assess*)) OR ((relative* OR famil* OR parent* OR dependent* OR dependant* OR social-environment* OR guardian* OR social-network* OR social-circle* OR caregiver* OR carer OR carers) NEAR/5 (self-report* OR questionnaire* OR survey* OR assess* OR report*)))) AND (TS=(cohort* OR longitudinal* OR prospective* OR retrospective* OR cross-section* OR ((follow-up* OR outcome*) NEAR/2 long*-term*) OR ((national* OR population* OR general*) NEAR/2 survey*) OR (control* NEAR/2 stud*)) OR TI=(follow-up* OR long*-term* OR register* OR registry* OR registries*)) AND (TS=(Europe* OR Albania* OR Andorr* OR Armenia* OR Austria* OR Azerbaij* OR Balkan* OR Baltic-States* OR Estonia* OR Latvia* OR Lithuania* OR Belarus* OR Belgi* OR Benelux* OR Bosnia* OR Herzegovin* OR Bulgaria* OR Channel-Island* OR Croatia* OR Czech* OR England* OR France* OR French* OR German* OR Gibraltar* OR Britain* OR British* OR Greece* OR Greek* OR Hungar* OR Ireland* OR Irish* OR Isle-of-Man* OR Ital* OR Kosov* OR Liechtenstein* OR Luxembourg* OR Malta* OR Maltese* OR Moldova* OR Monac* OR Montenegr* OR Netherland* OR Dutch* OR Poland* OR Polis* OR Portug* OR Macedonia* OR Romania* OR Russia* OR San-Marin* OR Scandinavia* OR Denmark* OR Danish* OR Faroe* OR Finland* OR Finnish* OR Iceland* OR Norway* OR Norweg* OR Sweden* OR Swedish* OR Scotland* OR Scottish* OR Serbia* OR Slovakia* OR Slovenia* OR Spain* OR Spanish* OR Switzerland* OR Swiss* OR Ukraine* OR United-Kingdom* OR Vatican* OR Wales* OR Welsh* OR (Georgia* NOT (usa OR united-states)))) NOT DT=(Meeting Abstract OR Meeting Summary) AND LA=(English)

Cinahl

(MH Multiple Trauma OR MH Accidents, Traffic OR MH Wounds, Penetrating OR MH Wounds, Nonpenetrating OR ((MH Accidents OR MH Emergency Medical Services+) AND MH "Wounds and Injuries") OR MH Amputation, Traumatic OR MH Trauma Severity Indices OR T(((multipl* OR severe* OR massive* OR major OR penetrat* OR blunt*) N2 (trauma* OR injur* OR wound* OR burn*)) OR (trauma* N2 amputat*) OR multitrauma* OR multiinjur* OR multi-trauma* OR multi-injur* OR polytrauma* OR polyinjur* OR poly-trauma* OR poly-injur* OR barytrauma* OR baryinjur* OR bary-trauma* OR bary-injur* OR ((traffic* OR car OR motorcycle* OR injur* OR emergen*) N2 accident*) OR (helicopter* N2 (trauma* OR emergenc*)) OR (brain N2 trauma*) OR neurotrauma* OR neuro*-trauma*) OR (traum* N2 (injur* OR wound*)) OR AB(((multipl* OR severe* OR massive* OR major OR penetrat* OR blunt*) N2 (trauma* OR injur* OR wound* OR burn*)) OR (trauma* N2 amputat*) OR multitrauma* OR multiinjur* OR multi-trauma* OR multi-injur* OR polytrauma* OR polyinjur* OR poly-trauma* OR poly-injur* OR barytrauma* OR baryinjur* OR bary-trauma* OR bary-injur* OR ((traffic* OR car OR motorcycle* OR injur* OR emergen*) N2 accident*) OR (helicopter* N2 (trauma* OR emergenc*)) OR (brain N2 trauma*) OR neurotrauma* OR neuro*-trauma*) OR (traum* N2 (injur* OR wound*))) AND (MH Patient Reported Outcomes OR MH Quality of Life OR MM Job Re-Entry + OR MM Anxiety+ OR MM Fear+ OR MM Stress Disorders, Post-Traumatic+ OR MM Psychological Well-Being + OR MM Mental Health+ OR ((MH Job Re-Entry OR MH Anxiety OR MH Fear OR MH Stress Disorders, Post-Traumatic OR MH Psychological Well-Being OR MH Mental Health OR MH Family OR MH Family Support OR MH Family Role+ ) AND (MH Questionnaires OR MH Self Report)) OR TI(((patient* OR self OR relatives OR family OR parent* OR dependent* OR dependant* OR social-environment* OR guardian* OR social-network* OR social-circle* OR caregiver* OR carer OR carers) N2 report* N2 outcome*) OR prom OR proms OR (quality N2 life) OR qaly* OR short-form OR shortform OR sf8 OR sf12 OR sf20 OR sf36 OR sf-8 OR sf-12 OR sf-20 OR sf-36 OR ((return-to-work* OR anxiety OR anxious* OR fear OR stress* OR ptsd OR wellbeing OR well-being OR mental-health OR psycholog*) N10 (self-report* OR questionnaire* OR survey* OR assess*)) OR ((relative* OR famil* OR parent* OR dependent* OR dependant* OR social-environment* OR guardian* OR social-network* OR social-circle* OR caregiver* OR carer OR carers) N6 (self-report* OR questionnaire* OR survey* OR assess* OR report*))) OR AB(((patient* OR self OR relatives OR family OR parent* OR dependent* OR dependant* OR social-environment* OR guardian* OR social-network* OR social-circle* OR caregiver* OR carer OR carers) N2 report* N2 outcome*) OR prom OR proms OR (quality N2 life) OR qaly* OR short-form OR shortform OR sf8 OR sf12 OR sf20 OR sf36 OR sf-8 OR sf-12 OR sf-20 OR sf-36 OR ((return-to-work* OR anxiety OR anxious* OR fear OR stress* OR ptsd OR wellbeing OR well-being OR mental-health OR psycholog*) N10 (self-report* OR questionnaire* OR survey* OR assess*)) OR ((relative* OR famil* OR parent* OR dependent* OR dependant* OR social-environment* OR guardian* OR social-network* OR social-circle* OR caregiver* OR carer OR carers) N6 (self-report* OR questionnaire* OR survey* OR assess* OR report*)))) AND (MH Prospective Studies + OR MH Controlled Before-After Studies OR MH Cross-Sectional Studies OR MH Registries, Trauma OR TI(cohort* OR longitudinal* OR prospective* OR retrospective* OR cross-section* OR ((follow-up* OR outcome*) N2 long*-term*) OR ((national* OR population* OR general*) N2 survey*) OR (control* N2 stud*)) OR (follow-up* OR long*-term* OR register* OR registry* OR registries*) OR AB(cohort* OR longitudinal* OR prospective* OR retrospective* OR cross-section* OR ((follow-up* OR outcome*) N2 long*-term*) OR ((national* OR population* OR general*) N2 survey*) OR (control* N2 stud*)) OR (follow-up* OR long*-term* OR register* OR registry* OR registries*)) AND (MH Europe+ OR TI(Europe* OR Albania* OR Andorr* OR Armenia* OR Austria* OR Azerbaij* OR Balkan* OR Baltic-States* OR Estonia* OR Latvia* OR Lithuania* OR Belarus* OR Belgi* OR Benelux* OR Bosnia* OR Herzegovin* OR Bulgaria* OR Channel-Island* OR Croatia* OR Czech* OR England* OR France* OR French* OR German* OR Gibraltar* OR Britain* OR British* OR Greece* OR Greek* OR Hungar* OR Ireland* OR Irish* OR Isle-of-Man* OR Ital* OR Kosov* OR Liechtenstein* OR Luxembourg* OR Malta* OR Maltese* OR Moldova* OR Monac* OR Montenegr* OR Netherland* OR Dutch* OR Poland* OR Polis* OR Portug* OR Macedonia* OR Romania* OR Russia* OR San-Marin* OR Scandinavia* OR Denmark* OR Danish* OR Faroe* OR Finland* OR Finnish* OR Iceland* OR Norway* OR Norweg* OR Sweden* OR Swedish* OR Scotland* OR Scottish* OR Serbia* OR Slovakia* OR Slovenia* OR Spain* OR Spanish* OR Switzerland* OR Swiss* OR Ukraine* OR United-Kingdom* OR Vatican* OR Wales* OR Welsh* OR (Georgia* NOT (usa OR united-states))) OR AB(Europe* OR Albania* OR Andorr* OR Armenia* OR Austria* OR Azerbaij* OR Balkan* OR Baltic-States* OR Estonia* OR Latvia* OR Lithuania* OR Belarus* OR Belgi* OR Benelux* OR Bosnia* OR Herzegovin* OR Bulgaria* OR Channel-Island* OR Croatia* OR Czech* OR England* OR France* OR French* OR German* OR Gibraltar* OR Britain* OR British* OR Greece* OR Greek* OR Hungar* OR Ireland* OR Irish* OR Isle-of-Man* OR Ital* OR Kosov* OR Liechtenstein* OR Luxembourg* OR Malta* OR Maltese* OR Moldova* OR Monac* OR Montenegr* OR Netherland* OR Dutch* OR Poland* OR Polis* OR Portug* OR Macedonia* OR Romania* OR Russia* OR San-Marin* OR Scandinavia* OR Denmark* OR Danish* OR Faroe* OR Finland* OR Finnish* OR Iceland* OR Norway* OR Norweg* OR Sweden* OR Swedish* OR Scotland* OR Scottish* OR Serbia* OR Slovakia* OR Slovenia* OR Spain* OR Spanish* OR Switzerland* OR Swiss* OR Ukraine* OR United-Kingdom* OR Vatican* OR Wales* OR Welsh* OR (Georgia* NOT (usa OR united-states)))) NOT PT(news OR congres* OR abstract* OR book* OR chapter* OR dissertation abstract*)
